# ISMB/ECCB 2023 proceedings

**DOI:** 10.1093/bioinformatics/btad319

**Published:** 2023-06-30

**Authors:** Yann Ponty, Sushmita Roy

**Affiliations:** Laboratoire d'Informatique de l'Ecole Polytechnique (CNRS UMR 7161), Institut Polytechnique de Paris, Palaiseau 91160, France; Wisconsin Institute for Discovery, University of Wisconsin-Madison, Madison, WI 53715, United States; Department of Biostatistics and Medical Informatics, University of Wisconsin-Madison, Madison, WI 53792, United States

This special issue of *Bioinformatics* serves as the proceedings of the 31st annual conference on Intelligent Systems for Molecular Biology (ISMB) co-organized with the 22nd European Conference on Computational Biology (ECCB). This conference took place on 23–27 July 2023, in Lyon, France. ISMB is the leading international forum for presenting new research results, disseminating methods and techniques, and facilitating discussions among leading researchers, practitioners, and students in the field. In addition, ISMB is the flagship conference of the International Society for Computational Biology (ISCB). Joining forces with ECCB, ISMB/ECCB2023 is the largest event of the computational biology community. Due to ongoing issues related to the COVID-19 pandemic, the ISMB/ECCB 2023 meeting was run as a hybrid conference, with online participants from all around the world complementing the on-site participants.

The papers published in this volume were selected from 336 submitted full length papers, featuring original research. The submitted papers were thoroughly reviewed with each paper receiving 4.04 reviews on average, for a total of 1358 reports. For the review purpose, the submitted manuscripts were assigned to one of 11 scientific areas according to authors’ preference and research topic, allowing for minor adjustments to avoid conflicts of interest. In addition to selecting an area, the authors could also designate a particular Community of Special Interest (COSI; Table 1) that would provide the best forum for presentation of their paper. The research areas covered a broad spectrum of topics (Table 2) and also included a special General Computational Biology area intended for submissions on emerging topics or for those manuscripts that did not fit well in other reviewing areas.

**Table 1.** COSI distribution of accepted ISMB 2023 Proceedings papers.

COSI	Number of Papers
3DSIG	4
BioOntologies	1
CAMDA	1
Education	1
EvolCompGen	5
Function	2
Varl	1
General Comp Bio	5
HitSeq	12
iRNA	2
Microbiome	6
MLCSB	8
NetBio	6
RegSys	5
TransMed	1

**Table 2.** Thematic areas of ISMB/ECCB 2023 proceedings talks. The table lists the Area Chairs for each theme, the number of reviewed papers, the number of accepted papers, and the acceptance rate for each area.

Area	Area Chairs	Submissions	Accept.	Accept. rate
Bioinformatics Education and Citizen Science	Nicola Mulder, University of Cape Town, South Africa	2	1	50%
	Jérôme Waldispühl, McGill University, Canada			
Bioinformatics of Microbes and Microbiomes	Robert Beiko, Dalhousie University, Canada	29	6	20%
	Hélène Touzet, CNRS, CRIStAL, France			
Biomedical Informatics	Zhiyong Lu, NIH, USA	58	10	17%
	Marinka Zitnik, Harvard University, USA			
Genome Privacy and Security	Hyunghoon Cho, Broad Institute (MIT/Harvard), USA	7	1	14%
	Gamze Gursoy, New York Genome Center, USA			
Evolutionary, Comparative and Population Genomics	Lars Arvestad, Stockholm University, Sweden	17	4	23%
	Céline Scornavacca, CNRS; Univ. Montpellier, France			
Genome Sequence Analysis	Rayan Chikhi, CNRS; Institut Pasteur, France	49	10	20%
	Rob Patro, University of Maryland, USA			
Macromolecular Sequence, Structure and Function	Sergei Grudinin, CNRS; Inria Grenoble, France	48	6	12%
	David H Mathews, Uni Rochester, USA			
Regulatory and Functional Genomics	Jian Ma, Carnegie Mellon University, USA	54	8	14%
	Marcel Schultz, University of Frankfurt, Germany			
Systems Biology and Networks	Tijana Milenkovic, University of Notre Dame, USA	52	10	19%
	Bo Wang, Univ Toronto, Canada			
General Computational Biology	Mohammed El-Kebir, University of Illinois, USA	20	4	20%
	Lenore Cowen, TUFTS University, USA			
Equity-focused Research	Casey Greene, University of Colorado Anschutz, USA	0	0	–
	Ran Blekhman, University of Chicago, USA			

The reviewing of the submissions was overseen by the Senior Program Committee (SPC), which included the Proceedings Chairs (this editorial’s authors) and Area Chairs (AC, listed in Table 2). Several of the ACs were nominated by COSIs or by the ISMB Steering Committee. Members of the SPC were responsible for recruiting the Program Committee. Reviewers (Program Committee Members and subreviewers) judged the papers based on the novelty of computational approaches, relevance of biological questions, importance of biological insights, clarity of presentations, expected impact, compatibility with the conference format and, most importantly, correctness, completeness, and reproducibility of the studies. After submitting their reviews, the reviewers had the opportunity to discuss the papers and refine the scores. The ACs facilitated these discussions, oversaw the review process and made sure reviews were detailed and consistent with the overall decision. Final acceptance decisions were made in agreement with the entire SPC.

Throughout the reviewing process, we followed a stringent policy of guarding conflicts of interest. Submissions that had any association to an Area Chair were reassigned to a different area. Care was taken not to assign papers to reviewers with a perceived conflict either (e.g. co-authorship or same institution). The definition of conflict was defined as broadly as possible—including collaborators (present and past few years), same institution (present, past few years, planned future moves), family relations, advisees/advisors, as well as any personal conflicts that could cause the appearance of conflict of interest, or that could genuinely interfere with objective reviewing. Finally, as Proceedings Chairs, we refrained from submitting papers to the conference.

Among the 336 submissions, 60 were accepted for presentation at ISMB/ECCB 2023 and publication in the proceedings, conditioned on revisions having properly addressed the comments of the reviewers. In a few cases, the authors had to be reminded to release their source code alongside their manuscript, or clarify some aspects of their evaluations. This year, all conditionally accepted papers were revised and subsequently judged to have properly addressed the concerns of the reviewers. They were subsequently accepted for the conference proceedings, resulting in a 17.9% acceptance rate overall. The acceptance rates for individual Areas are shown in Table 2. Accepted papers were assigned to COSIs based on the preferences of both authors and COSI organizers (Table 1).

We are deeply grateful to the Area Chairs, the 317 members of the Program Committee and the 368 subreviewers for their outstanding efforts in conducting thorough and timely reviews. Their contribution is at the core of the scientific quality of the conference. We also thank Steven Leard, Diane Kovats, and Seth Munholland for their support, guidance, and handling logistical questions and all the other members of the ISMB Steering Committee for their expert advise and supervision. We also thank the team at Oxford University Press for producing this special proceedings volume.

We finally thank all the authors for submitting their work. These proceedings would not be possible without the scientific ingenuity of the contributors of all the papers. We recognize that, despite our best efforts, the selection process is necessarily imperfect, and some outstanding work will have been missed. Nonetheless, we hope that all authors received helpful feedback on their work. Finally, we want to thank all the keynote speakers, presenters, and all conference participants.

Thank you all for making this meeting possible and the entire ISMB/ECCB community to continue to thrive.

